# Change in surgical practice amidst COVID 19; example from a tertiary care centre in Pakistan

**DOI:** 10.1016/j.amsu.2020.04.035

**Published:** 2020-05-01

**Authors:** Roshan-e-Shahid Rana, Muhammad Hammad Ather, Syed Ather Enam

**Affiliations:** a Aga Khan University Hospital, Pakistan; b Department of Surgery, Aga Khan University hospital, Pakistan

**Keywords:** COVID-19, Surgical practice

Surgical care is an integral component of any healthcare system and its continuous provision is essential for both elective and emergent cases. However, operating rooms (OR) are a high-risk zone for infection transmission. The surgical specialties and anesthetist are equally exposed. They often have to perform emergency surgeries in uncertain circumstances with patients COVID status undefined. This calls for immediate actions to maintain a balance between adequate provision of surgical services and preventing transmission along with judicious use of resources.

As of 22^ND^ April, there are 9749 confirmed cases, 209 deaths and 2156 recoveries from COVID 19 in Pakistan [1]. Country's first confirmed case of corona was diagnosed and treated in our hospital, which is a large tertiary care hospital in the largest city of the country. In the mid of March when this pandemic was gradually gripping our country, first surgery resident in our hospital was tested positive, quickly followed by 2 more positive doctors in department of surgery. Contact tracing lead to 29 surgery residents and interns being quarantined from three specialties; which was around 20% of the workforce. That was the first hit to the department and happened at a very early phase of the pandemic evolution in our country. This alerted the hospital authorities and called for aggressive strategies. In addition to the general preventive measures, department's leadership in liaison with each sub-specialty leads devised certain strategies in a quick, coordinated and efficient way to cope up with the pandemic. The main focus at that time was generic and directed at decreasing the exposure to healthcare providers, keeping sufficient manpower reserves and maintaining the quality of care. Unless, like most medical conditions, there are defined strategies or guidelines to cope with the pandemic, we were all learning from the experience. We therefore felt the significance of sharing our experience important in coping with this disaster. In retrospect, most of the recommendations were based on American College of surgeons' guidelines that was released at around the sametime [2].

1 **NEW PROVISION FOR BOOKING OF SURGICAL PROCEDURES AND OPERATING ROOM UTILISATION:** In mid-March, operating room was completely shut down for all sorts of elective and semi-elective cases, only urgent and emergent cases were allowed. In view of the fact that certain conditions and diseases may not be immediately life threatening but delay may have long term consequences, in particular cancers and caner related surgeries. Department in roughly two weeks time removed restriction to allow semi elective cases to be performed. Since semi-elective is a vague category and encompasses various procedures, for each specialty a list of allowable semi-elective procedures was prepared by surgical subspecialty heads and shared with operating room management to facilitate the process. A stringent criterion was followed, where in such cases were screened through a process. This include identification of patients at high risk of COVID-19 infections, approval by section head/service line chief followed by anesthetist approval (Fig. 1). Before finalizing, each list was discussed in a meeting between operating room (OR) leads including surgeon, anesthesiologists and nursing manager a day prior. Each specialty was assigned specific operating days and operating rooms for semi-elective cases. The number of OR functioning at any particular day were also reduced to 7 from normal 17 operating rooms; 5 for semi-elective cases and 2 for emergencies. A separate OR suite (normally used for orthopedic surgeries) which is located adjacent but away from the main operating area was dedicated for suspected or confirmed cases of COVID 19. This suite was designed recently in 2015 as a state of the art facility with laminar flow ceilings, individual temperature and humidity control, and High-efficiency particulate air (HEPA) filters.

**Fig. 1:**
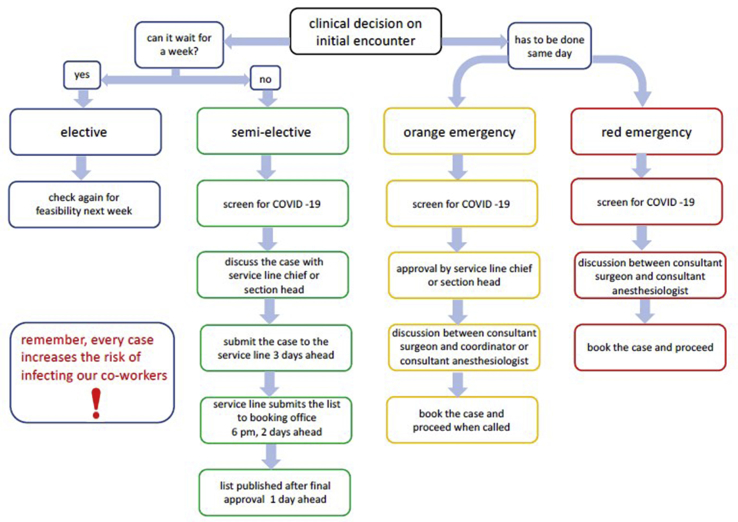
Process flow for surgical procedures.
2 **PROTOCOL FOR COVID SCREENING OF OR CASES:** All OR cases were screened by a three-item questionnaire at the time of booking and again 24 h prior to procedure. It included following questions: 1) Any symptoms of cough, runny nose, shortness of breath, sore throat in last 14 day, 2) history of travel (patient or family members) within last 14 days, 3) Contact with any COVID positive or suspected COVID patient. For semi-elective cases in case of any of these items being positive the case was re-assessed and necessary actions were taken after discussing with Infectious disease experts (Fig. 1). For emergency/urgent cases that were screened positive, the provision was to perform it in COVID designated OR following strict PPE.
3 **OUTPATIENT CLINICS:** Though the number of clinics in each specialty was cut down but at no time the clinics were on a complete shut down. Tele-clinics were also introduced from beginning of April; which before this pandemic were non-existent in our hospital. To decrease exposure of team members, the residents, interns and other medical officers were exempted from these clinics.
4 **DUTY ROSTER CHANGES:** In view of a sudden loss of work force, due to exposure quite a few of the residents and interns were quarantined, some drastic changes were made in the resident/interns duty roster. Each specialty was split into two teams with each team working on alternate weeks. Even on working week the duties were assigned in a way that only minimum number of required residents were on the floor. As an example if Team A and B has four residents each, two residents did alternate 24 h shift for a week. This was done to minimize the exposure and to keep sufficient manpower reserves for coverage in case of patient surge.
5 **ACADEMIC SESSIONS:** University stopped all classes and clinical activities for the medical/nursing students. Gathering of more than 5 was prohibited, which impacted administrative meetings and academic activities. However, within the first week of lockdown department decided to reconvene all academic sessions including grand round, Journal clubs, Tumor boards etc, albeit all online via Zoom™. It provided a portal for learning and also kept the team members connected.
6 **WELLNESS SESSIONS FOR PSYCOLOGICAL WELL BEING:** The significance of psychological well-being was recognized early on and steps were taken to address issues emanating from lockdown, all of a sudden very low clinical activity for some and extremely high and stressful activity for other health care providers. Frequent informal meetings were conducted by chair with faculty and residents to have their input, to know their feelings and also to provide them moral support. Department in collaboration with Psychiatry also offered short wellness sessions for residents.

It's been almost four weeks now that these strategies have been implemented one after the other and so far in department of surgery no major corona crisis has happened. Those who were positive and those who were exposed have now joined back work. Department right now aims to continue this scheme at least for this month.

Pakistan stands among lower middle-income countries with a weak healthcare infrastructure. It lacks significantly in key healthcare indicators compared to international standards [3]. Countries health care budget allocation had always been less than 1% of its GDP. The healthcare budget allocation for year 2019-20 was 11058 million, which is 20.4% lower than the budget estimates of 2018-19.[4] According to WHO global health workforce statistics the Physician to population ratio of Pakistan, is 0.97 per 1000 people. As per WHO estimates adequate coverage with primary care interventions requires at least 2.5 medical staff per 1000 people [5]. Country also struggles with the number of specialists, according to statistics by Pakistan medical and Dental Council the number of specialists registered in country till September 2019 were 46222 which is almost one fourth of GPs with basic degree only [6]. In this vulnerable mile lieu of health care system, any unusual burden bears the potential of massive health system crises. So strategies that cut down exposure are of paramount importance.

It is not possible to curtail the surgical care for a long time hence, a plan that could gain a balance between services and exposure was essential. Our department planned and implemented these strategies in a span of few days with continuous review for any shortcomings and with a constant reminder to team members, “every case increases the risk of infecting our co-workers.”

## Ethical approval

Doesn't involve patients and hence doesn't require ethical approval.

## Sources of funding

None.

## Declaration of competing interest

None to declare.

## Author contribution

Roshan-e-Shahid Rana: Conceptualisation, initial draft of manuscript, final proof reading and editing, reference search.

Hammad Ather: Conceptualisation of the whole change process within department, originally designed the process flow, final approval.

## Registration of research studies

Not applicable.

## Guarantor

Roshan-e-Shahid.

## Consent

Not applicable.

## Provenance and peer review

Not commissioned, Editor reviewed.
